# Neutrophil-to-Lymphocyte Ratio Is a Useful Marker for Predicting Histological Types of Early Gastric Cancer

**DOI:** 10.3390/jcm10040791

**Published:** 2021-02-16

**Authors:** Sho Yasui, Tomoaki Takata, Yu Kamitani, Yukari Mae, Hiroki Kurumi, Yuichiro Ikebuchi, Akira Yoshida, Koichiro Kawaguchi, Kazuo Yashima, Hajime Isomoto

**Affiliations:** Division of Gastroenterology and Nephrology, Faculty of Medicine, Tottori University, Tottori 683-8504, Japan; n9k_htke@yahoo.ne.jp (S.Y.); yukamitani@aol.com (Y.K.); yuuchanfront@gmail.com (Y.M.); kurumi_1022_1107@yahoo.co.jp (H.K.); ikebu@tottori-u.ac.jp (Y.I.); akirayoshida1021@yahoo.co.jp (A.Y.); koichiro@tottori-u.ac.jp (K.K.); yashima@tottori-u.ac.jp (K.Y.); isomoto@tottori-u.ac.jp (H.I.)

**Keywords:** endoscopic submucosal dissection (ESD), neutrophil-to-lymphocyte ratio (NLR), platelet-to-lymphocyte ratio (PLR), gastric cancer

## Abstract

Background: The indications for endoscopic submucosal dissection (ESD) for gastric cancer are based on preoperative histological assessment; however, examination of tissue biopsy is not always reliable as only a limited portion of the lesion can be obtained. The neutrophil-to-lymphocyte ratio (NLR) and platelet-to-lymphocyte ratio (PLR) are markers of inflammatory response and are potentially associated with the grade of malignancy in gastric cancer. We aimed to investigate the association between NLR and PLR and the histology of gastric cancer. Methods: This study included 218 patients who underwent ESD for gastric cancer. The relationship between NLR/PLR and histological diagnosis was investigated. Results: Patients with adenocarcinomas showed significantly higher NLR and PLR than those with adenomas (*p* < 0.001 and *p* < 0.05, respectively). Further, patients with undifferentiated adenocarcinoma showed a significantly higher NLR (*p* < 0.05) than those with differentiated adenocarcinoma. Conclusion: This study suggests that NLR could be a useful marker for assessing early gastric cancer.

## 1. Introduction

Gastric cancer is one of the most common gastrointestinal cancers and the third leading cause of cancer-related deaths worldwide [1]. Currently, a variety of treatment strategies are available for patients with early gastric cancer. Endoscopic submucosal dissection (ESD) is widely accepted and used as a treatment option for early gastric cancer. ESD is superior to conventional endoscopic mucosal resection in terms of removal of larger or ulcerated lesions in an en bloc manner and is more effective in preventing residual lesions and local recurrence.

Tissue biopsy is usually performed before ESD because histological evaluation is necessary in deciding the indication for ESD. However, only a portion of the lesion can be assessed by tissue biopsy; thus, specimens obtained by ESD sometimes contain histological types that are not diagnosed by tissue biopsy. In fact, in some cases, the histological types by tissue biopsy differ from the preoperative diagnosis, and this is related to an increased risk of endoscopic noncurative resection [2]. Undifferentiated-type components mixed with differentiated-type early gastric cancer have also been reported to be a risk factor for endoscopic non-curative resection [3]. It is important to accurately assess the histological type of early gastric cancer before performing ESD. Therefore, it would be of great impact to look for a new diagnostic method that supports conventional histological assessment.

The neutrophil-to-lymphocyte ratio (NLR) and platelet-to-lymphocyte ratio (PLR) are representative blood markers of the systemic inflammatory response [4] and high NLR/PLR have been shown to be significantly associated with poor prognosis in a variety of malignancies [5,6,7,8]. Additionally, a close relationship between the systemic inflammatory response and tumor progression has been demonstrated in some types of cancers [9,10]. Preoperative assessment of NLR has been found to be clinically relevant in predicting tumor progression and prognosis of surgically resectable gastric cancers [11]. We hypothesized that NLR and PLR are associated with the histological types of early gastric cancer and can be useful markers for determining the indication for ESD. The purpose of this study was to investigate the correlation of NLR and PLR with the histological type of early gastric cancer resected by ESD.

## 2. Materials and Methods

### 2.1. Study Population

This study included 218 patients who underwent ESD for gastric lesions between January 2017 and December 2019. The indications for ESD were based on the Gastric Cancer Treatment Guidelines of the Japanese Gastric Cancer Association [12]. Patients with systemic inflammation, multiple primary cancers, hematologic dysfunction, corticosteroid medication, and repetitive ESD within 3 months were excluded from this study. Those with gastric lesions other than adenomas or adenocarcinomas were also excluded. Patient characteristics, including age, sex, size of the lesion, laboratory data, and histological diagnosis, were collected from their medical records. This study was conducted in accordance with the principles of the Declaration of Helsinki and was approved by the ethical committee of the Tottori University Hospital (approval number: 1508A024). Written informed consent was obtained from all patients.

### 2.2. Clinical and Laboratory Findings

Laboratory findings before the ESD procedure were recorded. NLR was calculated as by dividing the neutrophil count by the lymphocyte count (Neu/Lym), and PLR was calculated by dividing the platelet count by the lymphocyte count (Plt/Lym). Patients with histologically proven adenocarcinoma was classified into two groups according to the histological types: a) the differentiated group comprised tubular adenocarcinoma well differentiated type (tub1), moderately differentiated type (tub2), and papillary adenocarcinoma (pap); b) the undifferentiated group comprised poorly differentiated adenocarcinoma solid type (por1), poorly differentiated adenocarcinoma solid type (por2), and signet-ring cell carcinoma (sig).

### 2.3. Statistical Analysis

Variables are expressed as mean ± SD or median (range). Differences between each group were analyzed using the Chi-square test for categorical variables, Student’s *t*-test for normally distributed variables, and the Mann–Whitney U test for non-normally distributed variables. The distribution of continuous variables was evaluated using the Kolmogorov-Smirnov test. StatFlex (version 7.0, for Windows, Artec, Osaka, Japan) and GraphPad Prism (version 7.0 for Windows, GraphPad Software, San Diego, CA, USA) were used for statistical analysis. A two-tailed *p*-value of <0.05, was considered significant.

## 3. Results

### 3.1. Patient Characteristics

**Figure 1:**
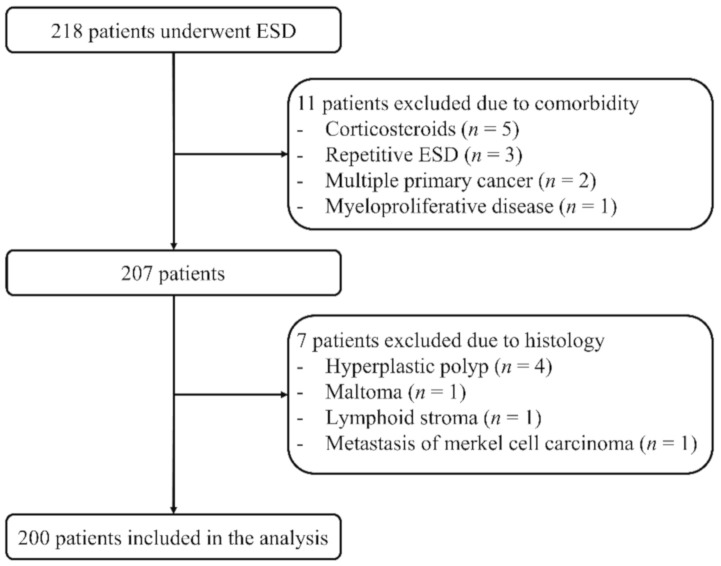
Study design. Among the 218 patients that were included in this study, 200 were analyzed and 18 were excluded.

Among 218 patients, 11 patients (five for corticosteroids, three for repetitive ESD, two for multiple primary cancer, and one for myeloproliferative disease) were excluded due to comorbidities. Of the remaining 207 patients, seven patients were further excluded from the analysis (four for hyperplastic polyps, one for maltoma, one for lymphoid stroma, and one for metastatic lesion). As a result, 200 patients were included in the analysis (Figure 1). Patient characteristics are summarized in Table 1.

### 3.2. Differences in NLR and PLR between Benign and Malignant Tumors

**Figure 2:**
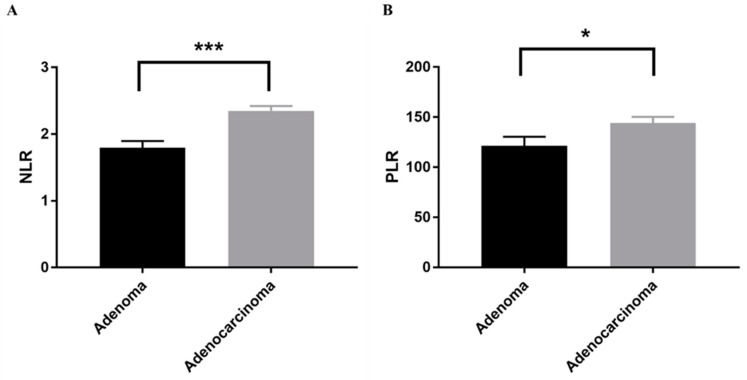
Comparison between adenoma and adenocarcinoma group. Quantitative analysis of (**A**) NLR and (**B**) PLR in the adenoma group and the adenocarcinoma group. Bars indicate average ± SEM. * *p* < 0.05 (Mann-Whitney U test); *** *p* < 0.001 (Student’s *t*-test). NLR, neutrophil-to-lymphocyte ratio; PLR, platelet-to-lymphocyte ratio.

In order to analyze the differences in NLR and PLR between patients with benign and malignant lesions, the patients were classified into an adenoma group and an adenocarcinoma group (Table 1). NLR and PLR were significantly higher in patients with adenocarcinomas than in those with adenomas (*p* < 0.001 and *p* < 0.05, respectively) (Figure 2).

### 3.3. Differences in NLR and PLR between Histological Types

**Figure 3:**
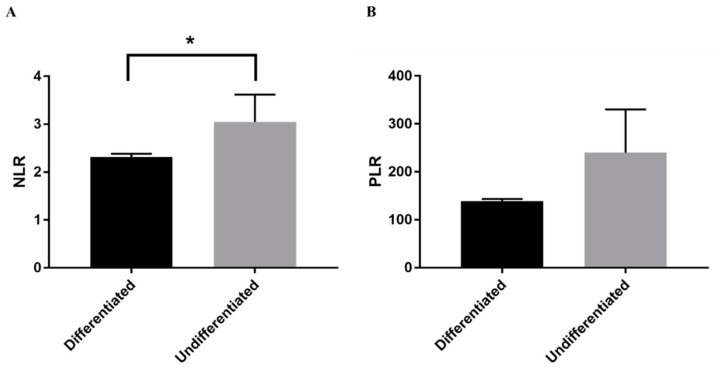
Comparison between histological types. Quantitative analysis of (**A**) NLR and (**B**) PLR in differentiated and undifferentiated adenocarcinoma. Bars indicate average ± SEM. * *p* < 0.05 (Student’s *t*-test). NLR, neutrophil-to-lymphocyte ratio; PLR, platelet-to-lymphocyte ratio.

We further investigated the differences in the NLR and PLR between the histological types. Patients with undifferentiated adenocarcinoma were significantly younger (*p* < 0.05) and showed a significantly higher NLR (*p* < 0.05) than those with differentiated adenocarcinoma (Table 2); however, there was no significant difference in PLR between both groups (Figure 3). Receiver operating curve analysis was performed to determine the cut-off value of NLR for diagnosing undifferentiated adenocarcinoma. As a result, NLR of 3.0 is the cut-off with a sensitivity of 76.3% and a specificity of 62.5%.

## 4. Discussion

In this study, we found that NLR and PLR were significantly higher in patients with adenocarcinoma. In addition, patients with undifferentiated adenocarcinoma had a higher NLR than those with differentiated adenocarcinoma. This study suggests that NLR could be a useful marker for estimating the histological type of early gastric cancer prior to ESD.

ESD for early gastric cancer is considered when the size and the histological type of the cancer matches the following conditions: differentiated mucosal cancers without ulcerative findings regardless of tumor size, differentiated mucosal cancers with ulcerative findings < 30 mm, differentiated minute (<500 μm from the muscularis mucosa) submucosal invasive cancers < 30 mm, and undifferentiated mucosal cancers without ulcerative findings < 20 mm with no lymphatic or vascular involvement [12]. The histological type of the lesion is an important factor in determining the indication for ESD. Preoperative tissue biopsy and endoscopic findings using magnifying endoscopy are conventional methods used to determine whether ESD should be used. In this study, we showed that NLR and PLR could be useful indicators for estimating the histological type of early gastric cancer.

NLR and PLR are ratios of two types of blood cell counts and are concise and simple markers. Neutrophils and platelets increase during inflammation [13,14], while lymphocytes decrease during inflammation in autoimmune diseases [15]. Infection and inflammatory responses have been demonstrated to be associated with many types of tumors, including gastric cancer. The inflammatory response is an important factor in the microenvironment of tumor cells [16,17]. The inflammatory response has been implicated in lymphopenia, neutrophilia, and thrombocytosis. The lymphocyte response also plays an important role in the immune response and is largely responsible for the inhibition of cancer progression [18]. A systemic inflammatory response associated with gastric cancer has been demonstrated [19], and this inflammatory response has been implicated in the prognosis of gastric cancer [20]. Inflammation-based markers, such as NLR and PLR have been shown to be poor prognostic factors in various solid tumors [5,6]. Some reports suggest that NLR and PLR are useful predictors of advanced operable gastric cancer and advanced gastric cancer treated with chemotherapy [21,22,23]. It has also been reported that increased NLR and PLR values are associated with higher tumor, node, and metastasis stage and are superior to conventional tumor markers such as carcinoembryonic antigen and carbohydrate associated antigen 19-9 for the early diagnosis of gastric cancer [24]. To the best of our knowledge, this is the first study to investigate the usefulness of NLR and PLR in predicting the histological type of early gastric cancer.

In this study, the preoperative NLR and PLR were higher in patients with adenocarcinoma than in those with adenomas. In addition, the undifferentiated group had a higher NLR than the differentiated group. Undifferentiated-type early gastric cancer was associated with a higher frequency of lymph node metastasis and invasive growth than differentiated-type early gastric cancer [25]. Therefore, we speculated that patients with adenocarcinomas have a higher NLR and PLR compared to those with adenoma. Furthermore, patients with undifferentiated-type early gastric cancer had a higher NLR than those with differentiated-type early gastric cancer. In this study, we propose that the cut-off value of NLR above 3.0 suggests the histological type of early gastric cancer as undifferentiated-type or undifferentiated-type components mixed with differentiated-type.

This study had several limitations. It is well known that *Helicobacter pylori* infection affects the NLR and PLR due to gastritis [26,27]. In this study, we also examined the history of *Helicobacter pylori* infection and background gastric mucosa, however there were no significant differences in the NLR and PLR of those patients that had *Helicobacter pylori* infection and those that were not infected. This was a retrospective study, and the number of subjects was small. Therefore, a prospective study with a larger number of patients is needed to cautiously verify the results of this study and to confirm the suitability of NLR and PLR as indicators for inferring the histological type of early gastric cancer.

## 5. Conclusions

In conclusion, NLR and PLR are putative indicators of the histological type of early gastric cancer that allow us to predict the indications for ESD and post ESD treatment strategies in patients with early gastric cancer.

## Figures and Tables

**Table 1:** Patients’ characteristics.

Parameter	Total	Adenoma	Adenocarcinoma	*p*-Value
Number	200	36	164	
Age, years	73.5 ± 8.8	72.2 ± 8.2	73.8 ± 8.9	ns
Sex, male/female	156/44	28/8	128/36	ns
Lesion size, cm^2^	2.3 (0.1–42.0)	2.4 (0.2–13.3)	2.3 (0.1–42.0)	ns
White blood cell count, /×10^3^μL	5.7 ± 1.5	5.9 ± 1.4	5.6 ± 1.5	ns
Neutrophil count, /×10^3^μL	3.5 ± 1.1	3.4 ± 1.0	3.5 ± 1.1	ns
Lymphocyte count, /× 10^3^μL	1.7 ± 0.6	2.0 ± 0.6	1.6 ± 0.5	<0.001 ^a^
Platelet count, /×10^3^μL	210 ± 61	224 ± 70	207 ± 59	ns
CRP, mg/dL	0.1 (0–3.6)	0.1 (0–2.4)	0.1 (0–3.6)	ns
NLR	2.2 ± 0.9	1.8 ± 0.6	2.3 ± 0.9	<0.001 ^a^
PLR	125 (40–844)	115 (50–353)	128 (40–403)	<0.05 ^b^

CRP, C-reactive protein; NLR, neutrophil-to-lymphocyte ratio; PLR, platelet-to-lymphocyte ratio. ^a^ Student’s *t*-test; ^b^ Mann-Whitney U test; ns, not significant.

**Table 2:** Characteristics in patients with adenocarcinoma.

Parameter	Differentiated	Undifferentiated	*p*-Value
Number	156	8	
Age, years	74.1 ± 8.5	67.1 ± 13.6	<0.05 ^a^
Sex, male/female	122/34	6/2	ns
Lesion size, cm^2^	2.3 (0.1–42.0)	1.7 (1.0–5.4)	ns
White blood cell count, /×10^3^μL	5.6 ± 1.5	5.2 ± 2.2	ns
Neutrophil count, /×10^3^μL	3.5 ± 1.1	3.4 ± 1.7	ns
Lymphocyte count, /×10^3^μL	1.6 ± 0.5	1.3 ± 6.3	ns
Platelet count, /×10^3^μL	206 ± 57	220 ± 94	ns
CRP, mg/dL	0.1 (0–3.6)	0.2 (0–1.4)	ns
NLR	2.3 ± 0.9	3.1 ± 1.6	<0.05 ^a^
PLR	128 (40–403)	149 (63–844)	ns

CRP, C-reactive protein; NLR, neutrophil-to-lymphocyte ratio; PLR, platelet-to-lymphocyte ratio. ^a^, Student’s *t*-test; ns, not significant.

## Data Availability

The data presented in this study are available on reasonable request from the corresponding author.
